# A Novel Virtual Planned-Orthodontic-Surgical Approach for Proportional Condylectomy in Condylar Hyperplasia

**DOI:** 10.3390/jcm14030752

**Published:** 2025-01-24

**Authors:** Stefania Perrotta, Emanuele Carraturo, Vincenzo D’Antò, Björn Ludwig, Tecla Bocchino, Luigi Angelo Vaira, Giacomo De Riu, Rosa Valletta, Pasquale Piombino

**Affiliations:** 1 Department of Neurosciences, Reproductive Sciences and Oral Sciences, Division of Orthodontics, University of Naples “Federico II”, Via Pansini 5, 80131 Naples, Italy; vincenzodanto@gmail.com (V.D.); tecla.bocchino2011@gmail.com (T.B.); valletta@unina.it (R.V.); 2 Department of Neurosciences, Reproductive Sciences and Oral Sciences, Division of Maxillofacial Surgery, University of Naples “Federico II”, 80131 Naples, Italy; emanuele.c2971995@gmail.com; 3 Department of Orthodontics, Institute of Odontology, Saarland University, 66421 Homburg, Germany; bludwig@kieferorthopaedie-mosel.de; 4 Sahlgrenska Academy, University of Gothenburg, 405 30 Gothenburg, Sweden; 5 Private Practice of Orthodontics, 56841 Traben-Trarbach, Germany; 6 Maxillofacial Surgery Unit, Department of Medicine, Surgery and Pharmacy, University of Sassari, 07100 Sassari, Italy; lvaira@uniss.it (L.A.V.); gderiu@uniss.it (G.D.R.); 7 Maxillofacial Surgery Unit, Caserta Hospital “Sant’Anna e San Sebastiano”, Via Palasciano, 81100 Caserta, Italy; pasquale.piombino@gmail.com

**Keywords:** condylar hyperplasia, condylectomy, virtual surgical planning, 3D printing, orthodontics first

## Abstract

**Background/Objectives:** Condylectomy is a delicate and intricate procedure commonly employed in the management of temporomandibular joint (TMJ) disorders, osteochondromas, condylar hyperplasia, hemimandibular hyperplasia, and other pathologies affecting the condylar region. The advent of surgical cutting guides has introduced a new dimension to condylectomy procedures as they enable surgeons to plan and execute precise cuts with a heightened level of accuracy. In the literature already exists cases of cutting guide-based condylectomy, but they only depend on the mere mirroring procedure in virtual planning, which has accuracy limitations because it does not consider asymmetry of peri-condylar structures at the level of the ramus, body, and mandibular angle. **Methods:** CAD-CAM orthodontic preparation through the NEMOFAB Software was performed to correct the canting of the occlusal plane, following the “orthodontic first” technique. The same software was used for VSP of the surgical cutting guide to perform the condylectomy, basing not to the mere mirroring of the opposite side but considering the whole condylar-TMJ-glenoid fossa structure. **Results:** At 6 months follow-up, the patient showed good occlusion and an almost totally recovered lower third symmetry as median-upper and lower interincisive lines coincide with each other and with the chin median. A good occlusal and masticatory outcome was obtained. The joint structure was preserved with remodeling of the glenoid cavity caused by the presence of the joint disc, which was preserved during surgery. **Conclusions**: The goal of this study is to propose a method of therapeutic management of condylar hyperplasia that benefits from accurate pre-operative orthodontic treatment (orthodontics first) to maximize the results of proportional condylectomy, reducing post-operative orthodontic care as well as any need for any adjuvant orthognathic surgery. A new virtual surgical planning method is also proposed for creating a cutting guide that not only takes advantage of the mirroring technique to accurately calculate the amount of condyle to be cut but also considers the entire condyle–TMJ complex to perform a condylectomy that is more precise.

## 1. Introduction

One of the aspects that makes maxillofacial surgery a very complex discipline is the usual need to operate surgically in very confined spaces and near noble anatomical structures [1,2]. The need to operate safely and the concomitant development of increasingly innovative technologies has meant that these technologies have entered the operating table of maxillofacial surgery operative units all over the world [3]. Surgical cutting guides are the perfect example of this. They have revolutionized various aspects of maxillofacial surgery, providing precision and accuracy in procedures such as condylectomy [4]. Condylectomy, the surgical removal of the mandibular condyle, is a delicate and intricate procedure commonly employed in the management of temporomandibular joint (TMJ) disorders, osteochondromas, condylar hyperplasia, hemimandibular hyperplasia, and other pathologies affecting the condylar region [5,6]. Traditional condylectomy techniques require a high level of surgical expertise and often involve intraoperative estimation of anatomical landmarks. However, the advent of virtual surgical planning (VSP) has introduced a new dimension to all TMJ procedures. VSP facilitates precise osteotomies and mandibular reconstructions in TMJ ankylosis treatment showing high accuracy in translating virtual plans to surgical outcomes; customized TMJ replacements using VSP and 3D-printed guides ensure accurate placement of prostheses with excellent post-operative precision. These guides, often designed based on pre-operative imaging such as computed tomography (CT) scans, enable surgeons to plan and execute precise cuts with a heightened level of accuracy [7,8]. The literature has shown the role of surgical cutting guides in enhancing the safety and efficacy of condylectomy, emphasizing their potential to optimize outcomes, reduce surgical time, and minimize post-operative complications in the management of various condylar pathologies. Previous studies defined this new surgical method “proportional condylectomy” and even described a minimally invasive approach utilizing intraoral VSP-guided condylectomy [9,10]. The other methods previously described base condylectomy alone or with orthognatic surgery in order to improve the symmetry of the maxilla and also eventually correct any sagittal deformity. Condylectomy, by the way, was calculated only on the mere mirroring procedure in virtual planning, which has accuracy limitations because it does not consider asymmetry of peri-condylar structures at the level of the ramus, body, and mandibular angle [11,12]. In this report, a new CAD-CAM design method for the surgical cutting guide, particularly in the management of condylar hyperplasia and hemimandibular hyperplasia, is presented that may represent an innovation in the planning and treatment of condylar pathologies.

## 2. Materials and Methods

A 17-year-old female patient visited the Orthodontic and Gnathology Operative Unit in Federico II University Hospital in November 2022. She presented with a severe asymmetric face with chin deviation to the right side, a class III malocclusion on the left side, and Class I on the right side with canting of the occlusal plane. The patient underwent orthopantomography, RX telecranium, cone beam CT, and a 99mTC single photon emission computed tomography (SPECT), showing high activity in the condylar region. Hemimandibular hyperplasia was diagnosed, for which condylectomy was planned after pre-operative orthodontic treatment.

### 2.1. Pre-Operative Orthodontic Treatment

**Figure 1:**
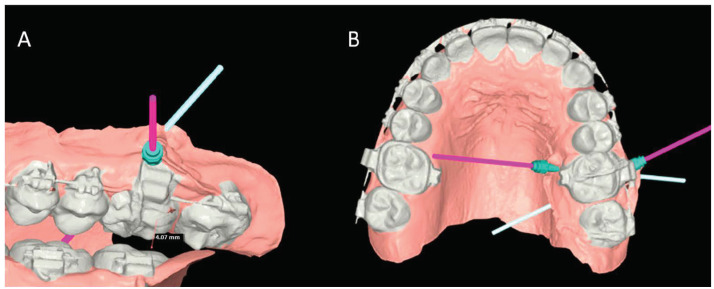
Vestibular (**A**) and Palatal (**B**) view of the virtual planning for the mini-screw positioning.

The orthodontic preparation was performed to correct the canting of the occlusal plane. The dental arches were bonded with a fixed multibracket appliance with an MBT prescription. A sequence of archwires was used, starting with 0.016 NiTi, 0.14 × 0.25 NiTi, and 0.19 × 0.25 NiTi arch sequences. Solid models of the mini-screws, teeth, and bone were imported and elaborated using NEMOfab software 2024 (NEMOTEC SL, Madrid, España) to generate CAD models based on the screw insertion direction at the infrazygomatic zone and palatal inter-radicular zones (Figure 1A,B).

**Figure 2:**
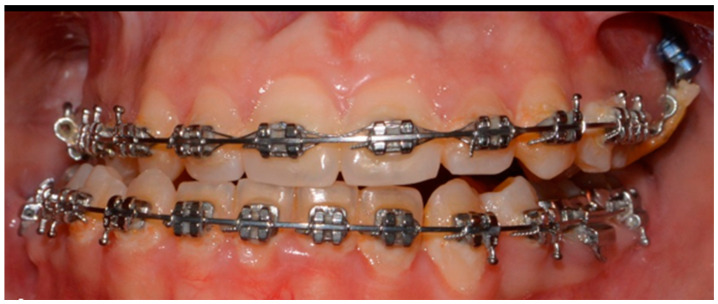
Orthodontics-first procedure, in frontal view.

While the leveling process continued, two mini-screws, 8 mm and 10 mm long, were inserted respectively in the vestibular and palatal inter-radicular safe zones. The intrusion with temporary anchorage devices (TADs) of 3 mm on the right maxillary side was virtually planned using CAD/CAM Nemofab 2024 software. Intrusive forces were applied to the left maxillary sector using elastic bands placed between a power arm installed on the vestibular infrazygomatic mini-screw and the palatal one, changed and reactivated every three weeks. Ultimately, after 8 weeks, a 0.19 × 0.25 SS arch was installed to increase the left maxillary intrusion (Figure 2).

After 12 months from the beginning of the treatment, the patient was then addressed to the Maxillofacial Surgery Operative Unit of the Federico II University Hospital for the surgery.

### 2.2. Virtual Planning

**Figure 3:**
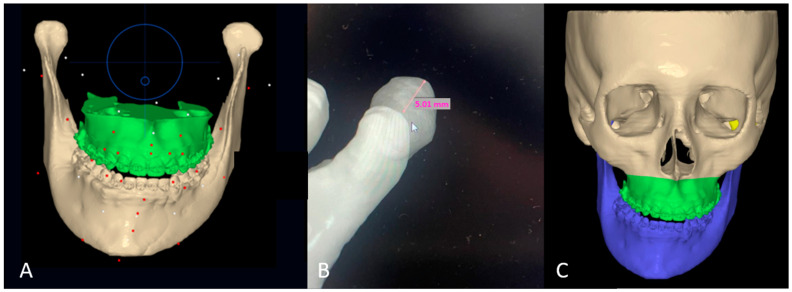
(**A**) Occlusion between the mandibular and maxillary arches, taking the latter as a reference; (**B**) Excess in vertical length in the hyperplastic condyle (5 mm); (**C**) tridimensional excess of the hyperplastic condyle obtained through Boolean operation from the lower mandibular border.

DICOM data from the CBCT were uploaded to the NEMOfab software. Taking the occlusal plane of the upper jaw in frontal view as a reference, previously corrected through the orthodontics-first method, the occlusal plane in frontal view has been evaluated. The mandibular arch is positioned in occlusion with the maxillary arch, and once uniform and punctiform contacts between the arches are obtained (Figure 3A), the virtual intrusion of the condyle within the glenoid fossa was evaluated by performing a Boolean operation between the glenoid cavity and hyperplastic condyle. Specifically, all three operations proper to Boolean operations (union, subtraction, and intersection) have been used to calculate both the comparison of the condyles and the subtraction of the gap in the cutting guide, i.e., the hole to insert the screw to attach the cutting guide to the condyle (Figure 3B). An arc is also drawn along the lower mandibular edge, having as its center the center of the chin. From this midpoint, a vertical line is drawn in a cranio–caudal direction, dividing the arc into two halves. The terminal points of the arc correspond to the mandibular gonion of the presumed healthy hemilateral. The verticals passing through these terminal points of the arc and parallel to the midline passing through the center of the arc/chin allow for a very accurate assessment of the differences in length between the healthy side and the hyperplastic side (Figure 3C).

**Figure 4:**
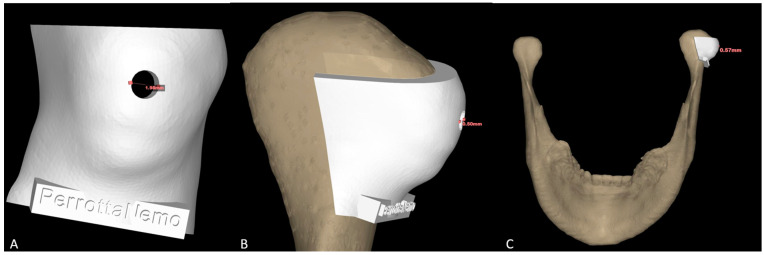
Condyle cutting guide in its frontal (**A**) and lateral (**B**,**C**) views.

Regarding the modeling of the cutting guide, through the “extrude” function offered by the NEMOfab software, the outer portion of the condyle in its lateral 2/3 was selected to design the cutting guide. A cylindrical dimension of about 2 mm in diameter by 0.57 mm in depth was then subtracted from this to draw the housing for the cutter entry (and the insertion of the subsequent screw) to clamp the cutting guide on the head of the patient’s hyperplastic condyle (Figure 4A–C).

**Figure 5:**
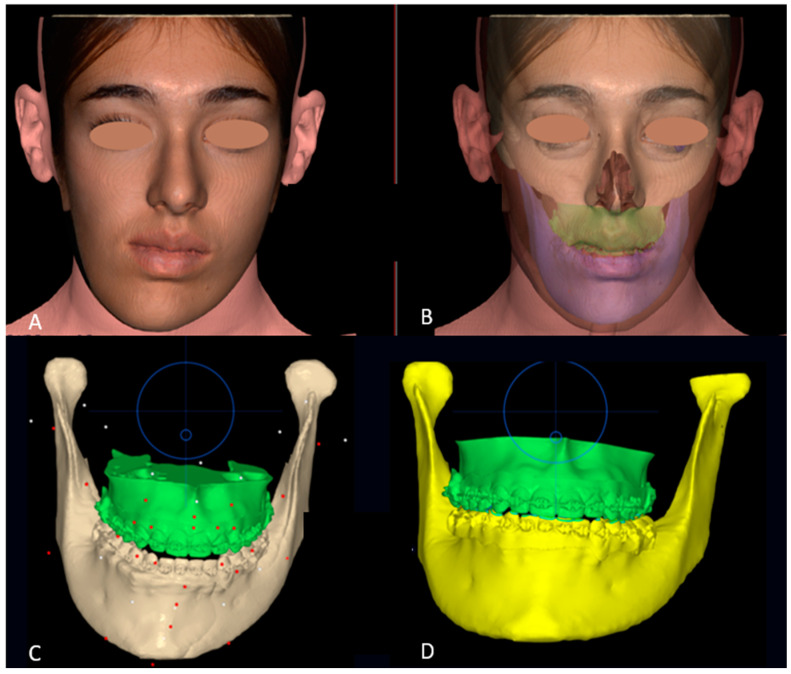
Pre-operative (**A**) and post-operative (**B**) virtual simulation of the facial soft tissues changes; Pre-operative (**C**) and post-operative (**D**) virtual simulation of the occlusal changes. Circles representing the joystick to handle selected items.

To intraoperatively test the accuracy of cutting guide planning, a stereolithographic model of the patient’s jaw was printed in-house. STL facial scans allowed the pre-operative evaluation of the aesthetic outcome of the procedure, which, at least virtually, demonstrates a recovery of the centrality of the lower third of the face (Figure 5A,B). Occlusal scans, on the other hand, allowed the evaluation of the functional outcome, ensuring the restoration of the correct occlusion (Figure 5C,D).

### 2.3. Surgical Procedure

**Figure 6:**
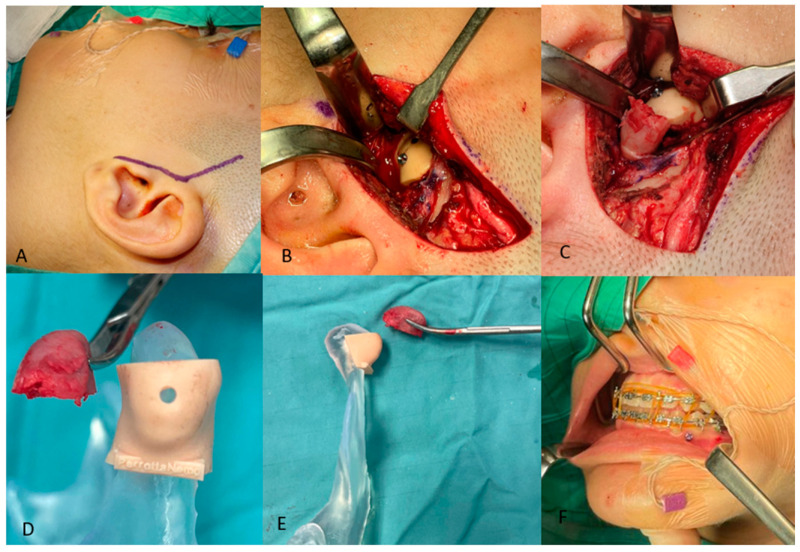
Condilectomy: Incision (**A**); insertion and securing of the cutting guide to the condyle head (**B**); osteotomy (**C**); comparison of the cutting guide upon the stereolithographic 3D model of the mandible with the condyle head removed in frontal (**D**) and lateral view (**E**); orthodontic set up and post-operative elastic contention (**F**).

The surgical procedure was performed under general anesthesia. After locoregional infiltration with carbocaine and a 2% adrenaline solution for hemostasis purposes, skin incision in the preauricular region, extending from the temporal region to the superior margin of the auricular lobe (Al-Kayat–Bramley incision) [13], was executed (Figure 6A). Soft tissues were dissected until reaching the superficial layer of the deep cervical fascia, and the adipose tissue contained in the deep temporal fascia was split. These elements were incised to reach the deep layer of the deep cervical fascia. By incising the periosteum of the malar arch, it was possible to reach the articular cavity. By dissecting the capsule at the level of the lateral temporomandibular ligament, it was possible to expose the head and neck of the condyle. The cutting guide was then inserted and secured to the head of the condyle with an 11 mm screw (Figure 6B). Finally, the osteotomy was performed through piezosurgery (Figure 6C). A 5 mm-long condyle stump was removed (Figure 6D,E). Nerve intraoperative monitoring (NIM) ensured that the facial nerve branches remained uninjured throughout the entire surgery.

### 2.4. Post-Operative Orthodontic Treatment

In addition to the mini-screws placed in the upper arch, a mini-screw was inserted in the lower arch between the canine and premolar, ipsilateral to the condylectomy, to use intermaxillary elastics from a skeletal anchorage (¼ 4 oz) with a Class III carrier to amplify the upward effect of the new condyle in the glenoid cavity and promote its remodeling (Figure 6F). Such skeletal elastics not only improve occlusion but also enhance chin centration in the frontal view. These elastics with a Class III carrier, from the lower arch to the upper arch (¼ 4 oz), are worn all day long. Orthodontic treatment at this stage is active and aims to improve occlusion and alignment, contributing to the improvement of facial aesthetics.

## 3. Results

**Figure 7:**
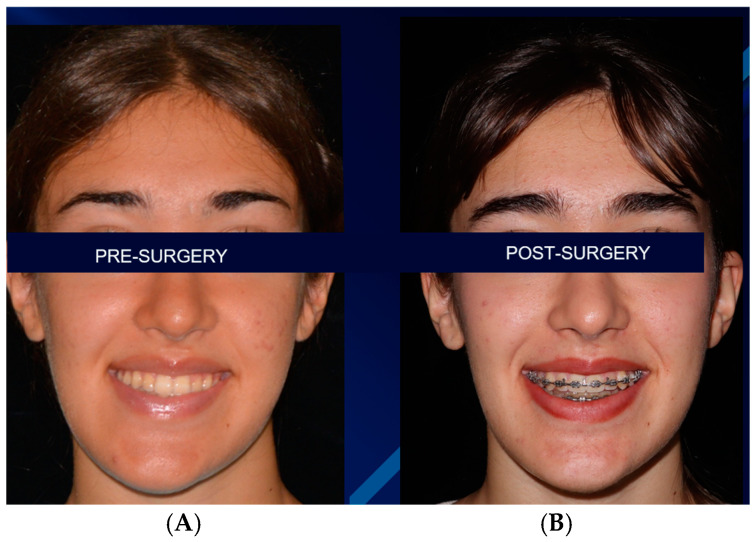
Pre-surgical (**A**) and 6 months follow-up post-surgical (**B**) appearances.

**Figure 8:**
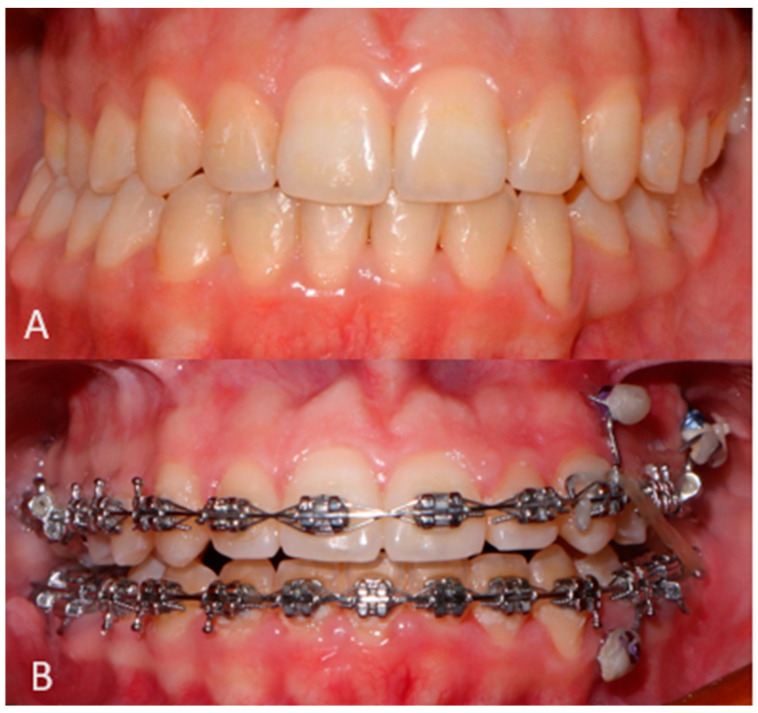
Pre-surgical (**A**) and 6 months follow-up post-surgical (**B**) occlusal appearance.

**Figure 9:**
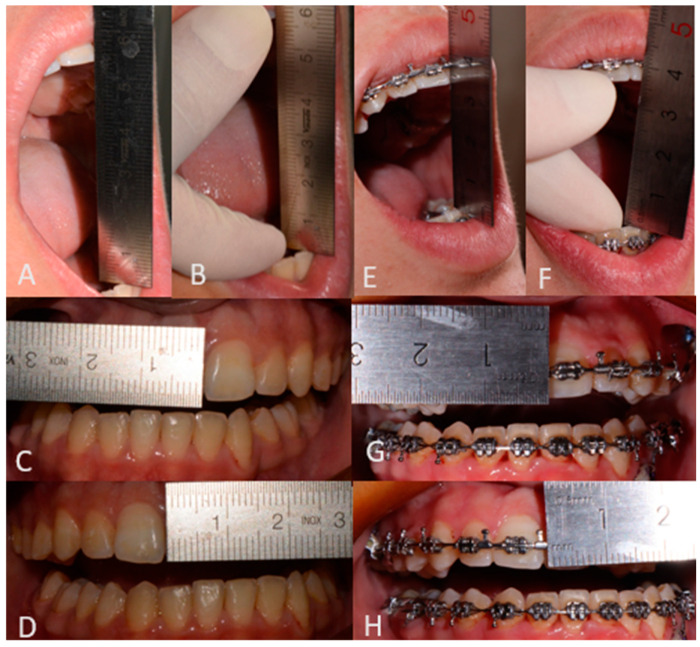
Pre-surgical occlusal assessment (**A**): Active opening 54 (+2) mm; (**B**): Passive opening 57 (+2) mm; (**C**): Right laterality 7 (−1) mm; (**D**): Left laterality 6 (+1) mm) and post-surgical occlusal assessment (**E**): Active opening 33 (+2) mm; (**F**): Passive opening 37 (+2) mm; (**G**): Right laterality 3 (−2) mm; (**H**): Left laterality 6 (+1) mm).

**Figure 10:**
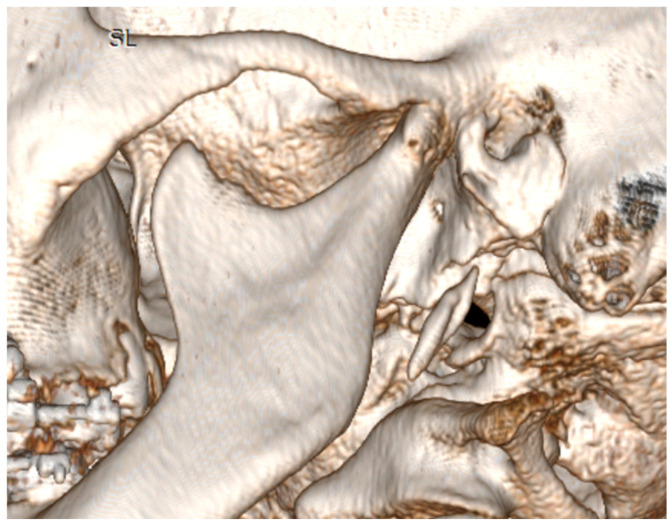
Post-operative appearance of the TMJ and glenoid fossa.

At 6 months follow-up, the patient showed good occlusion and an almost totally recovered lower third symmetry as median-upper and lower interincisive lines coincide with each other and with the chin median (Figure 7A,B and Figure 8A,B). With regard to occlusal and masticatory dynamics and kinetics, the patient had an excessive buccal opening (54 mm) prior to surgery, which often led to subluxations. In the post-operative period, correct occlusal dynamics were achieved, both in opening (33 mm) and lateral movements. (Figure 9). With regard to the status of the TMJ and the glenoid fossa in the post-operative period, as can be seen in Figure 10, the joint structure was preserved with remodeling of the glenoid cavity caused by the presence of the joint disc, which was preserved during surgery.

## 4. Discussion

Several classifications of condylar hyperplasia (CH) have been proposed over the years, each reflecting a deeper understanding of this condition’s complex nature [1,2]. In 1983, Delaire introduced a classification considering two biotypes of CH: one predominantly vertical and the other transverse. This early classification hinted at the multifaceted manifestations of CH, recognizing that growth patterns could significantly vary between individuals [13]. Expanding on this, Obwegeser and Makek in 1986 delineated three specific categories: hemimandibular hyperplasia (HH), hemimandibular elongation (HE), and a hybrid type blending characteristic of both HH and HE. This classification emphasized the distinction between horizontal and vertical growth patterns, contributing to a more nuanced understanding of CH’s clinical presentation [14]. Wolford, in 2014, further refined CH classification with a focus on the underlying pathologies and their manifestations. He identified four types: CH type 1 (CH1), which entails condylar and horizontal mandibular growth, subdivided into bilateral (CH1A) and unilateral (CH1B) cases; CH type 2, associated with osteochondroma leading to condylar enlargement (CH 2A) or exophytic osteochondroma (CH 2B); CH type 3, covering rare benign tumors originating from the mandibular condyle; and CH type 4, focusing on malignant tumors arising from the mandibular condyle [15]. These classifications share common observations regarding growth patterns. A predominantly horizontal growth pattern, as described by Delaire (transverse), Obwegeser and Makek (hemimandibular elongation), and Wolford (CH1B), typically leads to laterognathia. This condition often results in a posterior crossbite or edge-to-edge bite, lateral displacement on the non-affected side, class III malocclusion, prognathism, chin deviation, an open gonial angle without significant ramus discrepancy, a slight occlusal cant, and a depressed lip commissure on the affected side. Conversely, a vertical growth predominance, as outlined by Delaire (vertical), Obwegeser and Makek (hemimandibular hyperplasia), and Wolford (CH2A, excluding osteochondroma), manifests as an enlarged condylar head and neck, increased ramus height, and downward displacement of the mandibular body. Patients may exhibit vertical facial discrepancies, a canted occlusal plane, and a downward tilt of the lip commissure on the affected side (Table 1) [14,15,16].

To accurately diagnose and determine the appropriate treatment for CH, it is crucial to assess the pattern of growth activity and its progression. Techniques such as progressive radiographs, dental casts, and, especially, single photon emission computed tomography (SPECT) are employed to diagnose the pathology. SPECT/CT shows a high diagnostic validity for CH, significantly highlighting increased bone activity in the affected condyles compared to the contralateral side, aiding in confirming CH and guiding treatment options, as a difference in radionuclide uptake of 10% or more between the affected and the contralateral (non-affected) condyles is a significant indicator of active condylar hyperplasia [17,18]. As this classification refers to a bi-dimensional diagnosis, in recent years, a new trend in the literature has underscored the importance of utilizing CT and 3D reconstruction in distinguishing between various causes of facial asymmetry. Lopez et al., 2019, established a new method of diagnosis and classification through CT images that could be helpful in the correct management of facial asymmetry (FA). In their series, they have identified correctly six categories of FA: hemimandibular elongation, hemimandibular hyperplasia, hybrid hyperplasia, asymmetric mandibular prognathism, asymmetry of the glenoid fossa, and functional laterognathism, showing that the evaluation of the CT images and 3D reconstructions in patients with FA provided detailed information of the mandibular structure that is useful in comparing the differences between sides and in classifying the entities associated with FA [19]. Despite these commonalities, there is yet to be a standardized approach to diagnosing and treating CH, resulting in various surgical management protocols and algorithms being reported in the literature [20].

Commonly reported treatment methods include orthognathic surgery, exclusive surgical treatment of the temporomandibular joint (TMJ) through high or low condylectomy, or a combination of condylectomy and orthognathic surgery in one or two stages [21].

Condylectomy for hemimandibular and condylar hyperplasia is a surgical procedure designed to correct abnormal growth in the mandibular condyle. This procedure was comprehensively described by Bertolini et al. in 2001, detailing an early high condylectomy performed on a 12-year-old patient with hemimandibular hyperplasia, without the need for pre- or post-operative orthodontic treatment, yielding significant results [22]. This study underscored the crucial role of surgical intervention on the condyle for treating hemimandibular hyperplasia, and it also suggested the potential for integrating orthognathic surgery for a more comprehensive treatment, especially in patients who have reached skeletal maturity. After this, a paper by Wolford et al. in 2002 compared outcomes between two cohorts, the former undergoing orthognathic surgery alone and the latter receiving orthognathic surgery in conjunction with high condylectomy and disc repositioning. The second approach showed superior results. From these early studies onward, condylectomy has increasingly been considered the pivotal treatment in the management of condylar hyperplasia, whether or not accompanied by orthognathic surgery [23]. But what is the right amount of condyle to resect?

In the literature, there has been extensive debate about the necessity, differences, benefits, and outcomes of high condylectomy (removing between 3 and 5 mm of the condylar stump) versus low condylectomy (removing the ipsilateral condyle at the junction of the condylar head and neck while preserving the condylar neck), until Fariña et al. in 2015 introduced the concept of proportional condylectomy [24]. This approach involves a condylectomy where the lower limit of the osteotomy is marked based on pre-operative planning derived from diagnostic imaging (radiographs at the time), thus defining a condylectomy proportionate to the excess hyperplastic condyle based on the healthy counterpart condyle. Additionally, Fariña et al. demonstrated that, in an age-diverse cohort with an average age of 19, proportional condylectomy offers the advantage of avoiding the need for orthognathic surgery as an adjunct to condylectomy for achieving a favorable outcome, except in cases of concurrent craniodentofacial malformations [24]. Since then, the concept of proportional condylectomy has been heavily emphasized in the literature (but unfortunately, no uniform and clear distinction between the different methods is present in the literature). Historically, the size of condylar resection in the treatment of condylar hyperplasia was determined using two-dimensional cephalometric analysis. This method, as outlined by Mouallem et al., involved measuring the vertical distance between the mandibular angles and the supraorbital line on the coronal plane [25]. Similarly, Fariña et al. suggested obtaining differential measurements by drawing a line from the uppermost point of the condyle to the mandibular angle [24]. However, this approach cannot currently be considered valid due to the intrinsic limits of 2D imaging, especially in cases such as condylar hyperplasia. The growth center is not only localized within the condylar head but also distorts the entire anatomy of the mandibular body and the ramus–condyle unit. This distortion makes the determination of reference cephalometric points and lines challenging, rendering measurements for proportional condylectomy potentially unreliable. The advent of computed tomography (CT) and cone beam computed tomography (CBCT) marks a transformative shift in the diagnosis and treatment planning of condylar hyperplasia. These technologies enable three-dimensional visualization, enhancing the understanding of complex surgical challenges and, most importantly, they consent meticulous virtual surgical planning, allowing for a level of precision previously unattainable with conventional two-dimensional analyses. This progression highlights the critical role of advanced imaging techniques in refining the approach to diagnosing and managing condylar hyperplasia, underscoring the shift towards more sophisticated, accurate, and detailed assessment methods that leverage the full potential of 3D imaging and dedicated software for virtual surgical planning. In 2019, Sembronio et al. tackled seven cases of unilateral condylar hyperplasia using advanced techniques of virtual surgical planning. They used virtual planning to design and stereolithographically produce a cutting guide, enhancing the precision and replicability of the proportional condylectomy procedure [11]. This approach was complemented with elastic orthodontic treatment and, except for one patient, did not require any orthognathic surgery, falling within the concept of “adaptative condylectomy”, described by Nitzan [26]. The planning of this cutting guide utilized a method of mirroring and superimposition. This technique enables the creation of a virtual replica of the healthy half of the mandible, which serves as a reference to measure the excess vertical growth and guide the osteotomy on the affected condyle [11]. Although this method guaranteed great results and represents a milestone in the surgical planning of condyectomies, a possible improvement in this method is suggested in this study. Given that the mandibular condyle is a three-dimensional structure part of the temporomandibular joint (TMJ), resection/reduction of the condyle in a linear manner determined solely by mirroring should be questioned. As the glenoid fossa must be taken into account too, multiple factors are considered in our proposal (evaluation of the occlusal plane, positioning of the mandible in occlusion with the maxillary arch, movement into the glenoid fossa, and evaluation of the pericondylar structures), essential for greater accuracy and precision in decision-making for the osteotomy cut for condylectomy and for assessing a second surgical intervention on the mandibular body. Due to the differences observed in condylar hyperplasia and hemimandibular hyperplasia, mirroring alone could not be reliable for evaluating the osteotomy line in condylectomy, as it does not consider the aforementioned factors. Nevertheless, mirroring alone might remain valid in cases of purely condylar alteration. A second fundamental point has to be discussed: the relationship between condylectomy and occlusion is crucial, as the procedure not only aims to remove the hyperplastic bone tissue but also aims to improve or restore occlusal harmony and facial aesthetics. At present, there seems to be a gap in the literature on the merits of pre-operative occlusion assessment and management and its function in achieving a better post-operative outcome than isolated surgery. Therefore, this study proposes a condylectomy method that is strictly dependent on pre-operative orthodontic treatment according to orthodontics-first method described by Perrotta et al. in 2020 [27]. Patients with condylar hyperplasia or hemimandibular hyperplasia can gain great improvement in symmetry immediately after condylectomy if orthodontic preparation of the arches is performed first. Orthodontics-first approach involves increasing the inter-occlusal gap between the arches—specifically in the quadrant of the hemi-arch—ipsilateral to the excess defect by first creating alignment and leveling of the dental elements with archwire sequences (0.014 NiTi, 0.014 × 0.024 NiTi, 0.019 × 0.025 NiTi, and 0.019 × 0.025 SS). Then, an intrusion of the latero-posterior dental elements is created with the aid of 10 mm palatal and vestibular mini-screws (between the premolar and molar elements) from which an elastic chain is inserted, which is changed and activated every 3 weeks. In this way, before surgery, the patient will end up with a lateral open bite (ipsilateral to the defect). This orthodontically generated gap will allow the cut condyle to move up into the glenoid cavity enabling rotation of the mandible in the frontal plane, resulting in almost immediate resolution of the facial asymmetry. In this particular case, by calculating the excess condyle bone through the aforementioned method, an intrusion of the dental elements that should produce an inter-arch gap of 4–5 mm was estimated. As it is true that an intrusion no larger than 3 mm has been described in the literature, the gap of 4–5 mm has been considered corresponding to the amount needed to ensure a certain amount of arch exclusion and necessary for the rise of the condyle in the overall orthodontic-surgical planning. Obviously, a certain amount of recurrence after condylectomy is also considered in this context. Finally, it must be said that this type of treatment proposed in this paper can be applied for this specific condylar deformity, which provides for contained maxillary canting and complete absence of sagittal deformities of the jaws that would otherwise necessarily require concomitant or delayed orthognathic surgery.

## 5. Conclusions

This study proposes a method of therapeutic management of condylar hyperplasia that benefits from accurate pre-operative orthodontic treatment (orthodontics-first) to maximize the results of proportional condylectomy, reducing post-operative orthodontic care as well as any need for any adjuvant orthognathic surgery. A new virtual surgical planning method is also proposed for creating a cutting guide that not only takes advantage of the mirroring technique to accurately calculate the amount of condyle to be cut but also considers the entire condyle–TMJ complex to perform a condylectomy that is more precise. It has not yet been possible to extend the proposed treatment to a larger cohort, necessitating a case-control study to validate this method. However, this method may turn out to be useful in the management of condylar hyperplasia.

## Figures and Tables

**Table 1:** Comparison between the principal (bi-dimensional) classifications for condylar hyperplasia.

Delaire	Obwegeser and Makek	Wolford
Horizontal (transverse) pattern growth	Type I (Hemimandibular Elongation)	Type IA
		Type IB
Vertical pattern growth	Type II (Hemimandibular Hyperplasia)	Type IIA
	Type III (Combination of Type I and Type II)	Type IIB
		Type III
		Type IV

## Data Availability

The raw data supporting the conclusions of this article will be made available by the authors on request.

## References

[1] Pessoa Neto J.V., Cetira Filho E.L., Sampaio F.D., Mello M.J.R., Menezes Junior J.M.S. (2019). Removal of Foreign Bodies in Orbit-Zygomatic-Maxillary Complex. J. Craniofac. Surg..

[2] Abbate V., Romano A., AlQahtani A., Manfuso A., Hirt B., Castelnuovo P., Califano L. (2016). Midcheek endoscopic anatomy. Head Neck.

[3] Ayoub A., Pulijala Y. (2019). The application of virtual reality and augmented reality in Oral & Maxillofacial Surgery. BMC Oral Health.

[4] Haas Junior O.L., Fariña R., Hernández-Alfaro F., de Oliveira R.B. (2020). Minimally invasive intraoral proportional condylectomy with a three-dimensionally printed cutting guide. Int. J. Oral Maxillofac. Surg..

[5] Wolford L.M., Movahed R., Dhameja A., Allen W.R. (2014). Low condylectomy and orthognathic surgery to treat mandibular condylar osteochondroma: A retrospective review of 37 cases. J. Oral Maxillofac. Surg..

[6] Vásquez B., Olate S., Cantín M., Sandoval C., Fariña R., Del Sol M. (2016). Histopathological analysis of unilateral condylar hyperplasia: Difficulties in diagnosis and characterization of the disease. Int. J. Oral Maxillofac. Surg..

[7] Cascone P., Runci Anastasi M., Maffia F., Vellone V. (2021). Slice Functional Condylectomy and Piezosurgery: A Proposal in Unilateral Condylar Hyperplasia Treatment. J. Craniofac. Surg..

[8] Du W., Yang M., Liu H., Ji H., Xu C., Luo E. (2020). Treatment of hemimandibular hyperplasia by computer-aided design and computer-aided manufacturing cutting and drilling guides accompanied with pre-bent titanium plates. J. Craniomaxillofac. Surg..

[9] Si J., Zhang C., Tian M., Jiang T., Zhang L., Yu H., Shi J., Wang X. (2023). Intraoral Condylectomy with 3D-Printed Cutting Guide versus with Surgical Navigation: An Accuracy and Effectiveness Comparison. J. Clin. Med..

[10] Figueroa A.A., Harmon K.A., Arnold S., Xu H., Roy T., Reid R.R., Tragos C. (2024). The Role of Digital Surgical Planning and Surgical Guides in the Treatment of Unilateral Condylar Hyperplasia. J. Craniofac Surg..

[11] Sembronio S., Tel A., Costa F., Robiony M. (2019). An Updated Protocol for the Treatment of Condylar Hyperplasia: Computer-Guided Proportional Condylectomy. J. Oral Maxillofac. Surg..

[12] Han B., Wang X., Li Z., Yi B., Liang C., Wang X. (2018). Hemimandibular Hyperplasia Correction by Simultaneous Orthognathic Surgery and Condylectomy Under Digital Guidance. J. Oral Maxillofac. Surg..

[13] Al-Kayat A., Bramley P. (1979). A modified pre-auricular approach to the temporomandibular joint and malar arch. Br. J. Oral Surg..

[14] Delaire J., Gaillard A., Tulasne J.F. (1983). La place de la condylectomie dans le traitement des hypercondylies [The place of condylectomy in the treatment of hypercondylosis]. Rev. Stomatol. Chir. Maxillofac..

[15] Obwegeser H.L., Makek M.S. (1986). Hemimandibular hyperplasia--hemimandibular elongation. J. Maxillofac. Surg..

[16] Wolford L.M., Movahed R., Perez D.E. (2014). A classification system for conditions causing condylar hyperplasia. J. Oral Maxillofac. Surg..

[17] Yang Z., Reed T., Longino B. (2016). Bone Scintigraphy SPECT/CT Evaluation of Mandibular Condylar Hyperplasia. J. Nucl. Med. Technol..

[18] Hamed M., AlAzzazy M., Basha M. (2017). The validity of SPECT/CT in diagnosis of condylar hyperplasia. Egypt. J. Radiol. Nucl. Med..

[19] López D.F., Botero J.R., Muñoz J.M., Cárdenas-Perilla R., Moreno M. (2019). Are There Mandibular Morphological Differences in the Various Facial Asymmetry Etiologies? A Tomographic Three-Dimensional Reconstruction Study. J. Oral Maxillofac. Surg..

[20] Xu M., Chan F.C., Jin X., Xu J., Lu J., Zhang C., Teng L. (2014). Hemimandibular hyperplasia: Classification and treatment algorithm revisited. J. Craniofac. Surg..

[21] Niño-Sandoval T.C., Maia F.P.A., Vasconcelos B.C.E. (2019). Efficacy of proportional versus high condylectomy in active condylar hyperplasia—A systematic review. J. Craniomaxillofac. Surg..

[22] Bertolini F., Bianchi B., De Riu G., Di Blasio A., Sesenna E. (2001). Hemimandibular hyperplasia treated by early high condylectomy: A case report. Int. J. Adult Orthod. Orthognath. Surg..

[23] Wolford L.M., Mehra P., Reiche-Fischel O., Morales-Ryan C.A., García-Morales P. (2002). Efficacy of high condylectomy for management of condylar hyperplasia. Am. J. Orthod. Dentofac. Orthop..

[24] Fariña R., Olate S., Raposo A., Araya I., Alister J.P., Uribe F. (2016). High condylectomy versus proportional condylectomy: Is secondary orthognathic surgery necessary?. Int. J. Oral Maxillofac. Surg..

[25] Mouallem G., Vernex-Boukerma Z., Longis J., Perrin J.P., Delaire J., Mercier J.M., Corre P. (2017). Efficacy of proportional condylectomy in a treatment protocol for unilateral condylar hyperplasia: A review of 73 cases. J. Craniomaxillofac. Surg..

[26] Nitzan D.W. (2023). ‘Adaptable condylectomy’ for acquired facial asymmetry and malocclusion caused by temporomandibular joint condylar hyperplasia. Int. J. Oral Maxillofac. Surg..

[27] Perrotta S., Lo Giudice G., Bocchino T., Califano L., Valletta R. (2020). Orthodontics First in Hemimandibular Hyperplasia. “Mind the Gap”. Int. J. Environ. Res. Public Health.

